# Astrocyte-derived miR-124 impairs glioma cell volume regulation and migration by reducing Ca^2+^-dependent IK channel expression and activation

**DOI:** 10.1007/s00018-026-06217-x

**Published:** 2026-05-09

**Authors:** Myriam Catalano, Arianna Rinaldi, Angela Di Battista, Cristina Limatola, Luigi Catacuzzeno, Antonio Michelucci

**Affiliations:** 1 Department of Physiology and Pharmacology, Laboratory affiliated to Istituto Pasteur Italia, Sapienza University, Rome, Italy; 2 Department of Chemistry, Biology, and Biotechnology, University of Perugia, Perugia, Italy; 3 IRCCS Neuromed, Via Atinense 18, Pozzilli, 86077 IS Italy

**Keywords:** Astrocyte-derived extracellular vesicles, Glioblastoma, Glioma, miR-124, Intermediate-conductance Ca^2+^-activated K^+^ channel, VRAC, ER Ca^2+^ signaling, ERK1/2, RyR1, IP_3_R3, Cell volume regulation, Migration

## Abstract

Astrocytes play a key role in regulating glioma cell volume, a major determinant of tumor invasiveness. We previously reported that astrocyte-derived extracellular vesicles (ADEVs) transfer microRNA miR-124 to murine glioma cells, impairing cell volume regulation and reducing the functional expression of volume-regulated anion channel (VRAC). Here, we identify the intermediate-conductance Ca^2+^-activated K^+^ (IK) channel, the only volume-regulated K^+^ channel acting in concert with VRAC in GL261 glioma cells, as an additional indirect target of miR-124. Mechanistically, ADEV-derived miR-124 decreases the expression of ryanodine receptor type-1 (RyR1) and inositol 1,4,5-trisphosphate receptor type-3 (IP_3_R3), the principal endoplasmic reticulum (ER) Ca^2+^ release channels in these cells. This reduction in ER-mediated Ca^2+^ signaling markedly decreases ERK1/2 phosphorylation and IK channel gene transcription. Consistent with these molecular effects, fetal bovine serum-induced IK channel activation, driven by ER Ca^2+^ release, was strongly reduced by ADEVs and miR-124, and three-dimensional migration was impaired. Notably, combined pharmacological inhibition of IK and VRAC recapitulated the deficits in volume regulation induced by ADEVs and miR-124. Collectively, these findings show that ADEVs restrict glioma cell migration through a miR-124-dependent pathway that suppresses ER Ca^2+^ release, reduces ERK1/2 activation, and diminishes IK channel expression and function. Together with our previous in vivo evidence for ADEVs- and miR-124 -mediated targeting of VRAC, these results identify coordinated regulation of K^+^ and Cl⁻ channels as a mechanism by which astrocytes constrain glioma progression.

## Introduction

Glioblastoma (GBM) is the most aggressive primary brain tumor, characterized by rapid progression and extremely poor clinical outcomes [1–3]. A defining feature of GBM malignancy is the pronounced infiltrative behavior of tumor cells, which invade surrounding brain parenchyma, thereby preventing complete surgical resection and promoting inevitable recurrence. To migrate through the narrow extracellular spaces of the brain, glioma cells must undergo rapid and spatially restricted changes in cell volume and shape.

Ion channels play a central role in these volume-adaptive mechanisms by regulating osmolyte flux and the consequent osmotic movement of water across the plasma membrane [4, 5]. In particular, the volume-regulated anion channel (VRAC), which mediates Cl⁻ efflux through the swelling-activated Cl⁻ current (I_Cl, swell_), together with Ca^2+^-activated K^+^ (K_Ca_) channels, which provide the accompanying K^+^ flux, enables coordinated anion and cation efflux required for regulatory volume decrease (RVD) in GBM cells [6–9]. RVD is an evolutionarily conserved process that restores cell volume following osmotic swelling and is widely used as an experimental model to study cell volume regulation. Among K_Ca_ channels, the large-conductance (BK) and intermediate-conductance (IK) channels are overexpressed in human GBM cells and have been implicated in tumor cell migration and invasiveness [5, 10–13]. However, despite extensive molecular characterization of these channels, the upstream mechanisms that regulate their activity in the context of tumor invasion remain incompletely understood.

Increasing evidence indicates that the tumor microenvironment critically shapes GBM progression. Astrocytes, which constitute nearly half of all brain cells, establish extensive bidirectional communication with glioma cells and represent key regulators of tumor behavior [14]. A major mechanism mediating this cross-talk involves extracellular vesicles (EVs), including miRNA-rich exosomes, which function as potent intercellular signals within the GBM microenvironment capable of reprogramming the gene expression and phenotype of recipient cells. Numerous studies have shown that GBM-derived EVs remodel neighboring astrocytes into pro-tumorigenic effectors that promote tumor progression, invasion, and therapy resistance [15–20]. Conversely, naïve, non-tumor-primed astrocytes display potent anti-tumor activity [21], underscoring the functional plasticity of astrocytes and highlighting EV-mediated communication as a key regulatory mechanism in the GBM microenvironment.

We previously demonstrated that EVs released by non-tumor-primed astrocytes (ADEVs) hinder GBM progression in vivo and suppress invasion of GL261 glioma cells in vitro while impairing RVD [9]. Mechanistically, ADEVs deliver miR-124, which directly targets the VRAC subunit *LRRC8C*, thereby reducing channel activity and compromising cell volume recovery. Given that efficient RVD requires coordinated activation of VRAC and K_Ca_ channels, which together mediate Cl⁻ and K⁺ efflux [6, 7, 22–25], it remained unclear whether ADEVs also affect K_Ca_ channels and, if so, through which molecular mechanisms.

In the present study, we investigated the contribution of IK or BK channels to RVD in GL261 cells and examined whether ADEVs regulate their expression and function. Our results reveal that IK channels are the predominant functional K_Ca_ conductance in GL261 cells and play a crucial role in RVD. ADEVs suppress IK channel expression and activity through a miR-124-dependent mechanism that reduces ERK1/2 phosphorylation, a key regulator of IK channel gene (*KCNN4*) transcription, by downregulating the endoplasmic reticulum (ER) Ca^2+^ release channels RyR1 and IP_3_R3. Functional analyses indicate that RyR1 and IP_3_R3 cooperate to sustain ER Ca^2+^ signaling required for ERK1/2 activation and IK channel expression. Consistently, pro-migratory stimuli such as fetal bovine serum (FBS) fail to robustly activate IK channels in ADEV-treated or miR-124-transfected GL261 cells, and similar effects are observed upon pharmacological inhibition of ER Ca^2+^ release through RyRs and IP_3_Rs. This signaling cascade ultimately limits IK channel activation and markedly reduces three-dimensional cell migration. Notably, simultaneous inhibition of both IK and VRAC channels is required to suppress RVD to the same extent observed after ADEV treatment, confirming their cooperative contribution to volume regulation.

Together with our previous in vivo findings showing that ADEVs suppress glioma growth through VRAC inhibition [9], these results identify coordinated regulation of Cl⁻ and K⁺ channels as a key mechanism underlying the antitumor activity of ADEVs and miR-124 and highlight VRAC and IK channels as potential therapeutic targets for limiting GBM progression.

## Materials and methods

### Cell line and treatment

GL261 murine glioma cells were cultured in Dulbecco’s modified Eagle’s medium (DMEM; Thermo Fisher Scientific, Waltham, MA, USA) supplemented with 20% heat-inactivated, exosome-depleted fetal bovine serum (FBS; Thermo Fisher Scientific), prepared by ultracentrifugation at 100,000 x g for 18 h at 4 °C, followed by filtration through a 0.22-µm filter prior to use in cell culture, as previously described [26], 100 IU/mL penicillin G, 100 µg/mL streptomycin, and 2.5 µg/mL amphotericin B (all from Thermo Fisher Scientific). Cells were maintained at 37 °C in a humidified atmosphere containing 5% CO_2_. For transfection experiments, GL261 cells (1 × 10^5^ cells/mL) were seeded in 12-well plates and either mock-transfected or transfected with a miR-124 mimic (product ID MC10691, Thermo Fisher Scientific) using Lipofectamine™ 3000 reagent (Thermo Fisher Scientific) in Opti-MEM™ reduced-serum medium (Thermo Fisher Scientific) for 24 h, according to the manufacturer’s instructions. The 24-h incubation period was chosen because preliminary experiments demonstrated that this duration was sufficient to achieve maximal reduction in target protein expression; extending the incubation to 48 h did not further decrease expression, consistent with optimal miRNA-mediated regulation reported by the manufacturer. In selected experiments, GL261 cells were treated with the mitogen-activated protein kinase kinase (MEK) inhibitor PD98059 (30 µM; Sigma-Aldrich, St. Louis, MO, USA). All analyses were performed 24 h after treatment.

### Primary astrocyte cultures

Primary astrocytes were isolated from mixed glial cultures derived from cerebral cortices of postnatal day 0–2 (P0-P2) C57BL6/N mice. Cortices were minced and digested with 16 U/mL papain (Merck KGaA, Darmstadt, Germany) for 20 min at 37 °C. Cells were plated at 5 × 10^5^ cells/cm^2^ on poly-L-lysine hydrobromide-coated flasks (0.1 mg/mL; Merck KGaA) and cultured in growth medium containing 10% heat-inactivated exosome-depleted FBS. After 9–11 days, cultures were shaken for 2 hours at 37 °C to detach microglia, yielding purified astrocyte cultures. ADEVs were isolated as previously described [9].

### Transwell assay

Cell migration was assessed using Transwell chambers (24-well format, 8 μm pore size membrane; Sigma-Aldrich, Milan, Italy). GL261 cells were trypsinized, preincubated in chemotaxis medium (DMEM, 100 IU/mL penicillin G, 100 µg/mL streptomycin, 0.1% BSA, and 25 mM HEPES, pH 7.4) and seeded in the same medium in the upper chamber. In the lower chamber, 1% FBS was added as a chemoattractant. After incubation for 24 h at 37 °C, cells were fixed with ice-cold 10% trichloroacetic acid for 10 min and stained with a solution containing 50% isopropanol, 1% formic acid, and 0.5% Brilliant Blue R-250. Non-migrated cells on the upper membrane surface were removed gently with a cotton swab. Migrated cells on the lower surface were imaged and counted under a light microscope using a 40x objective.

### Patch-clamp electrophysiology

Whole-cell patch-clamp recordings were performed using either the dialyzed or perforated-patch configuration, depending on the specific experimental protocol. Currents and voltages were amplified using a HEKA EPC-10 amplifier (List Medical, Darmstadt, Germany) and analyzed with the *PatchMaster* software package (version 2_60, HEKA Elektronik) and *Microcal Origin 8.0*. Macroscopic currents were filtered at 3 kHz and sampled at 50 µs/point for on-line data collection. Patch pipettes (resistance 3–5 MΩ) were fabricated from borosilicate glass capillaries (OD 1.5 mm, ID 0.84 mm; World Precision Instruments) using a PUL-100 micropipette puller (World Precision Instruments). For all experiments, the same extracellular Ringer’s solution contained (in mM): NaCl 140 mM, KCl 5 mM, CaCl_2_ 2 mM, MgCl_2_ 2 mM, HEPES 5 mM and glucose 10 mM, (pH 7.40). The dialyzed whole-cell configuration was used for most experiments, including IK and BK current expression under isotonic conditions or their activation under hypotonic conditions. Access resistances ranged between 5 and 15 MΩ. The internal solution contained (in mM): KCl 155, EGTA-K 1, HEPES 5 and MgCl_2_ 1 (pH 7.20). The desired free Ca²⁺ concentration (400 nM, for maximal IK current activation) was obtained by adding the appropriate amount of CaCl_2_, calculated using the *WEBMAXC STANDARD* software. The 30% hypotonic solution was prepared by adding distilled water to the isotonic extracellular Ringer’s solution. The perforated-patch configuration was used to record the FBS-activated IK current. The internal pipette solution contained (in mM): K_2_SO_4_ 57.5, KCl 55, MgCl_2_ 5, and HEPES 10 (pH 7.2). Electrical access to the cytoplasm was achieved by adding amphotericin B (200 µM) to the pipette solution. Access resistances of 15–30 MΩ were achieved within 10–15 min from seal formation. For all experiments, octanol (1 mM) was included to block gap-junctional coupling and electrically isolate the patched cell [27]. The following pharmacological agents were used: 4-[(2-Butyl-6,7-dichloro-2-cyclopentyl-2,3-dihydro-1-oxo-1 H-inden-5-yl)oxy] butanoic acid (DCPIB)(Tocris Bioscience, Bristol, UK), a potent and recognized inhibitor of VRAC-mediated I_Cl, swell_, was dissolved in dimethyl sulfoxide (DMSO, Sigma-Aldrich, Milan Italy) at the stock concentration of 50 mM and used at different concentrations (in µM) in the recording solution: 0.1, 0.3, 1, 3, 10, 30. This concentration fully blocks VRAC as previously reported [6]. TRAM-34, a selective inhibitor of IK channels, dissolved in DMSO at the stock concentration of 10 mM and used at different concentrations (in µM) in the recording solution: 0.01, 0.1, 0.3, 1, 3. Paxilline and TEA, potent and selective blockers of BK channels were dissolved in DMSO and distilled water at the stock concentration of 10 mM and 3 M and used at the final concentrations of 1 µM and 3 mM, respectively. NS309, a potent activator of IK channels, was dissolved in DMSO at the stock concentration of 50 mM and used at the final concentration of 10 µM. The final DMSO concentration in all recording solutions did not exceed 0.1%. All reagents were freshly prepared each day and bath-applied using a gravity-driven perfusion system. Experiments were carried out at room temperature (18–22 °C).

### Videoimaging for cell volume measurements

Cell volume changes were assessed using time-lapse video imaging. Cells were perfused with an extracellular Ringer’s solution containing (in mM): NaCl 140, KCl 5, CaCl_2_ 2, MgCl_2_ 2, HEPES 5, and glucose 10 (pH 7.4). For these experiments, octanol was omitted from the solution. To induce hypotonic swelling, cells were exposed to a 30% hypotonic solution prepared as described in the *Patch-clamp electrophysiology* section. Cells were visualized through a 40x objective lens on an inverted microscope connected to a video camera (Axiocam/Cm1, Zeiss, Dublin, CA, USA). Images were acquired every 2 min over a total recording period of 60 min, saved as TIFF files, and analyzed using *ImageJ* software. Changes in cell volume were inferred from the relative projected cell area (A_rel_), calculated at each time point (A_t_) by normalizing to the baseline area (A_0_), defined as the average of the three measurements taken during isotonic conditions in the first 4 min. The extent of RVD was quantified 60 min after the onset of the regulatory response, shortly after the peak of swelling, using the formula: %RVD = (A_rel,peak_−A_rel,60 min_)/(A_rel,peak_)*100, where A_rel,peak_ and A_rel,60 min_ are the relative areas at the peak of cell swelling and at the end of the experiment, respectively [6, 7, 25]. In each experimental session, only cells maintaining a round shape throughout the experiment were included in the analysis. GL261 cells under control conditions (i.e., in the absence of pharmacological inhibition of IK and BK channels) were used as controls for the cells either treated with TRAM-34 (to block IK channels) or TEA (to block BK channels) and were recorded within the same experimental sessions. For each condition tested, at least one control was acquired in parallel on the same day, ensuring that all comparisons were conducted under identical conditions. Each experimental condition included a minimum of three biological replicates from independent cultures. Experiments were conducted at room temperature (18–22 °C).

### Western blot analysis

GL261 cells were lysed in RIPA buffer (50 mM Tris-HCl, 150 mM NaCl, 1% Triton X-100, 0.1% SDS, 1% deoxycholate, 2 mM EDTA, pH 7.5) supplemented with protease and phosphatase inhibitors (Sigma-Aldrich, Milan, Italy). Protein samples (20 µg per lane) were separated by 8.75% SDS-PAGE and transferred to nitrocellulose membranes (Amersham Protran, Sigma-Aldrich). Membranes were probed overnight at 4 °C with primary antibodies against phospho-p44/42 MAPK (Erk1/2) (Thr202/Tyr204) and total p44/42 MAPK (Erk1/2) (both 1:1000; Cell Signaling Technology, Boston, MA, USA). HRP-conjugated goat anti-rabbit IgG secondary antibodies (1:1000; Dako, Cernusco sul Naviglio, Italy) were used for detection. Signals were visualized using Amersham ECL Prime Western Blotting Detection Reagent (Cytiva, UK) and quantified by densitometric analysis with Quantity One software (version 4.6.6, Bio-Rad, Hercules, CA, USA).

### Gene expression analysis

The cells were lysed in Trizol reagent (Invitrogen, Milan, Italy) for RNA extraction, RNA was quantified and *retro*-transcribed using IScriptTM Reverse Transcription Supermix (Bio-Rad, Hercules, California, USA).

*Quantitative real-time PCR (RT-qPCR): g*ene expression was assessed using the I-Cycler IQ Multicolor RT-PCR Detection System (Bio-Rad, Hercules, California, USA) using SsoAdvanced Universal SYBR Green Supermix (Bio-Rad, Hercules, California, USA). The PCR protocol consisted of 40 cycles of denaturation at 95 °C for 30 s and annealing/extension at 58 °C for 30 s. The Ct values from each gene were normalized to the Ct value of *Gapdh*. Relative quantification was performed using the 2^−ΔΔCt^ method and expressed as fold increase. Primer sequences for mRNA analysis are listed below:

- *Ip3r3* FW: 5′-GGGCGCAGAACAACGAGAT-3′ and.
- RW: 5′-GAAGTTTTGCAGGTCACGGTT-3′.
- *Ryr1* FW: 5′-*CCGGGACACCCGGGGATTGC*-3′ and.
- RW: 5′-*GAGCACCGTGGCGCTGCACT*-3′.
- *Ryr2* FW: 5′-*ACGGCGACCATCCACAAAG*-3′ and.
- RW: 5′-*AAAGTCTGTTGCCAAATCCTTCT*-3′.
- *Ryr3* FW: 5′-*CGAGGGACTTGGGAATCGC6*-3′ and.
- RW: 5′-CTTGCAGTGCTCTGACAGATAA-3′.
- *Kcnn4* (IK) FW: 5′-GGCTGAAACACCGGAAGCTC-3′ and RW: 5′-CAGCTCTGTCAGGGCATCCA-3′;
- *Kcnma1* (BK) FW: 5′-TCTCAGCATTGGTGCCCTCGTAAT-3′ and RW: 5′-GTAGAGGAGGAAGAACACGTTGAA-3′;
- *Sos1* FW: 5’-GAGCCAAACACGAGAGACAC-3′ and RW: 5′-ATTCTGCACTGCTAGCACCA-3′;
- *Ras* FW; 5’-TGTGGATGAGTACGACC-3′; RW: 5’-ACGGAATCCCGTAACTC-3’.
- *Raf* FW: 5’-TCCAGGAGACCAAATTCCAG-3’; RW: 5’-GTGCAAGCATTGATGTCCTC-3’; *Gapdh* FW: 5′-TCGTCCCGTAGACAAAATGG-3′; RW: 5′-TTGAGGTCAATGAAGGGGTC-3′.

*Conventional PCR and electrophoresis gels: *Conventional PCR was performed using DreamTaq Green PCR Master Mix (Thermo Fisher Scientific, Waltham, MA, USA) and cDNA obtained as described above according to the manufacturer instructions. 1% agarose gel was prepared using Certified Molecular Biology Agarose (Bio-Rad, Hercules, California, USA) dissolved in 1X Tris/Acetic Acid/EDTA (TAE) buffer (Bio-Rad, Hercules, California, USA) by heating the solution in a microwave oven for 2–3 minutes. Ethidium bromide (10 mg/ml diluited 1:10,000, Euroclone S.p.A, Pero (MI), Italy) was added to the melted agarose, and it was immediately poured on a UV transparent gel casting tray fitted with a 10-well comb. The gel tray was placed on a horizontal electrophoresis system (Bio-Rad, Hercules, California, USA) and the electrophoresis chamber was filled with 1X TAE buffer to about 1 cm above the gel. 10 µl of each PCR sample was loaded into each well and electrophoresis was performed for 30’ and the power supply set to 100 V. A DNA size marker (Gene ruler 1 kB plus, Bio-Rad, Hercules, California, USA) was used.

### Statistical analysis

Statistical analyses were performed using Origin version 8.0 (OriginLab Corp., Northampton, MA, USA). Data are presented as mean ± SEM. Normality was assessed using the Shapiro-Wilk test. Comparisons between two independent groups were made using an unpaired two-tailed *t*-test, while comparisons against a theoretical value (unity) were analyzed using a one-sample *t*-test. For comparisons involving more than two groups, one-way ANOVA followed by Tukey’s *post hoc* test was applied. Exact *P* values are reported, and statistical significance was set at *P* < 0.05. Sample sizes (*n*) are indicated in the figure legends. All experiments were independently repeated at least three times to ensure reproducibility.

## Results

### IK channels are the only expressed K_Ca_ channels in GL261 cells and are essential for RVD

**Fig. 1:**
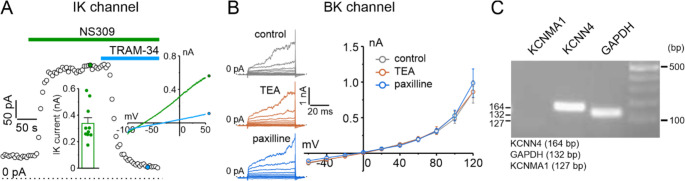
Expression and functional assessment of IK and BK channels in GL261 cells.(**A**) Representative time course of IK current (nA) at 0 mV recorded using voltage ramp protocols under control conditions, after application of 10 µM NS309 (dark green bar), and following inhibition with 3 µM TRAM-34 (cyan bar). *Insets*: (left) Average maximal IK current (nA) at 0 mV, calculated as the difference between NS309-activated and TRAM-34-inhibited currents (*n* = 10); (right) representative current traces evoked by voltage ramps from − 100 to + 50 mV (1 s duration; holding potential −40 mV) under NS309 (dark green trace) and NS309 + TRAM-34 (cyan trace) application. (**B**) Mean current-voltage (I-V) relationships recorded under control conditions (grey circles), in the presence of 3 mM TEA (dark orange circles), or 1 µM paxilline (blue circles) (*n* = 5). *Insets*: Representative current traces obtained using 50-ms voltage steps from −60 to +120 mV (20-mV increments; holding potential −40 mV) under control (grey traces), TEA (dark orange traces), or paxilline (blue traces) conditions. (**C**) Representative agarose gel electrophoresis of PCR amplification products from reverse-transcribed RNA extracted from confluent GL261 cell cultures (*n* = 4). Primers targeting *KCNMA1* (127 bp) encoding for BK channel and *KCNN4* (164 bp) encoding for IK channel were used. *GAPDH* (132 bp) served as aninternal control. Data are shown as mean ± SEM. Individual data points are reported for each experimental condition. All experiments were performed in at least three independent preparations

We previously reported that IK and BK channels cooperatively contribute to RVD in human U87-MG cells by facilitating the coordinated K^+^ and Cl⁻ efflux in conjunction with VRAC channels [7, 25]. To determine whether a similar mechanism operates in murine GL261 glioma cells, we first characterized the functional expression of K_Ca_ channels using whole-cell dialyzed patch-clamp recordings (Fig. 1A, B).

IK channel activity was isolated by pharmacologically blocking VRAC and BK channels with 10 µM DCPIB and 1 µM paxilline, respectively, and by clamping intracellular Ca^2+^ at 400 nM to selectively promote IK current activation. Recordings were performed in voltage-clamp mode using 1-s voltage ramps from −100 to +100 mV (holding potential, − 40 mV). Under these conditions, application of the IK channel activator NS309 (10 µM) elicited a prominent outward, voltage-independent current that was fully abolished by the selective IK channel inhibitor TRAM-34 (3 µM; Fig. 1A). Quantitative analysis revealed a mean TRAM-34-sensitive current of 337.6 ± 43.1 pA at 0 mV, demonstrating robust functional expression of IK channels in GL261 cells.

In contrast, BK channel activity was assessed using voltage-step protocols ranging from −60 to +120 mV (holding potential, −40 mV) in the constant presence of 10 µM DCPIB and 3 µM TRAM-34, and using 3 mM TEA or 1 µM paxilline to effectively suppress BK channel-mediated currents. Neither inhibitor altered outward current amplitudes at depolarized potentials nor affected the current-voltage (I-V) relationship (Fig. 1B), indicating the absence of functionally expressed BK channels in GL261 cells. These electrophysiological observations were corroborated by conventional end-point RT-PCR analysis of K_Ca_ channel gene expression (Fig. 1C). Consistent with the patch-clamp results, *KCNN4* transcripts, encoding IK channels, were abundantly expressed, whereas *KCNMA1* transcripts, encoding BK channels, were nearly undetectable, confirming the lack of BK channel expression at the mRNA level.

**Fig. 2:**
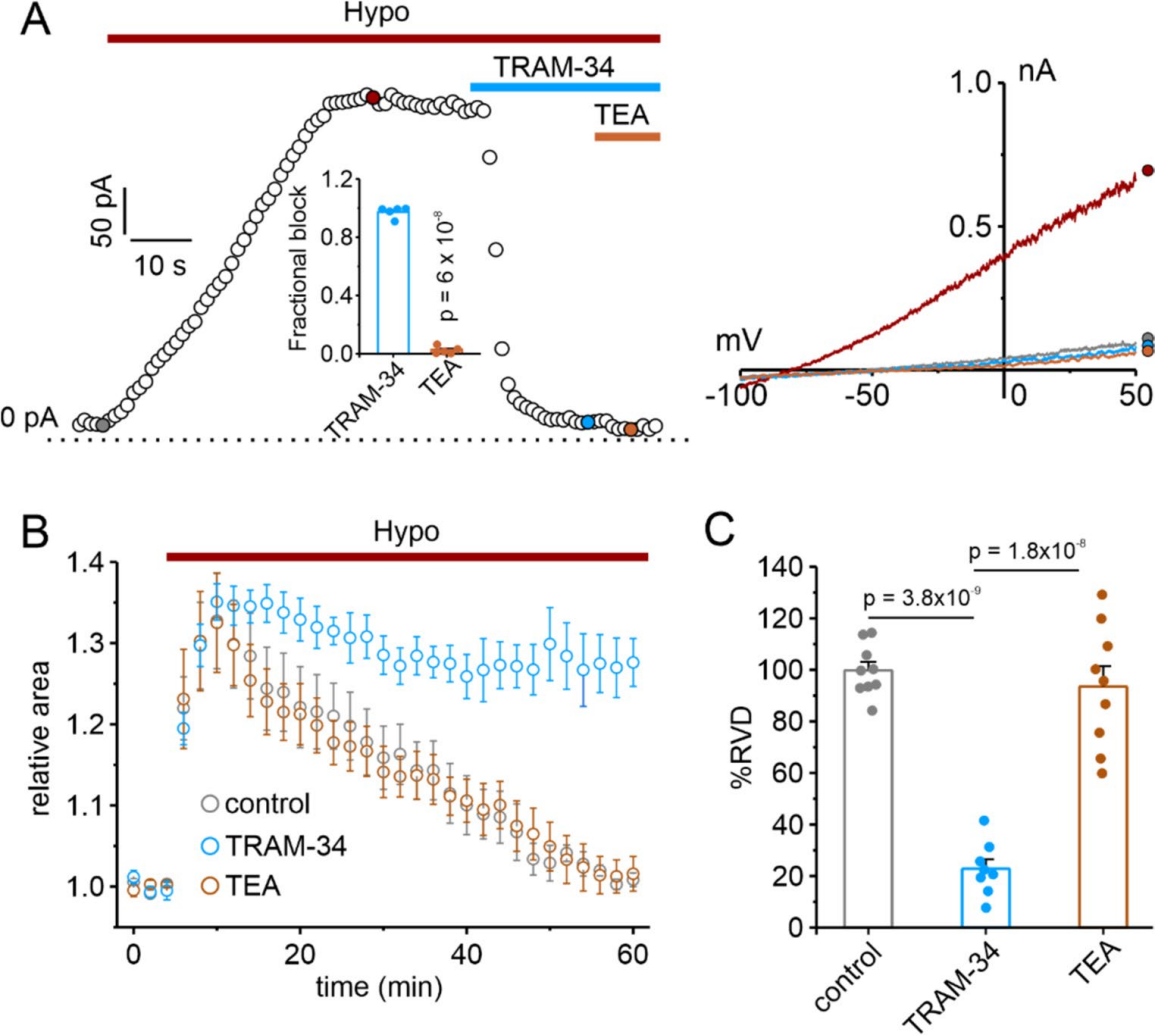
Evaluation of IK and BK channel contributions to RVD in GL261 cells.(**A**) *Left*: Representative time course of outward current (pA) at 0 mV during exposure to 30% hypotonic solution (red bar), in the presence of 10 µM DCPIB to block I_Cl,swell_, followed by sequential application of 3 µM TRAM-34 (cyan bar) and 3 mM TEA (dark orange bar). *Inset*: Average fractional inhibition of the hypotonic-activated current by TRAM-34 and TEA. *Right:* Representative current traces evoked by voltage ramps from −100 to +50 mV (1 s duration; holding potential − 40 mV) under isotonic conditions (grey trace), after hypotonic challenge (red trace), and following addition of TRAM-34 (cyan trace) or TRAM-34 + TEA (dark orange trace). (**B**) Time course of RVD measured as changes in relative cell area during exposure to 30% hypotonic solution (red bar) in control cells (grey circles; *n* = 9), or in cells treated with 3 µM TRAM-34 (cyan circles; *n* = 7) or 3 mM TEA (dark orange circles; *n* = 9). **(C)** Average RVD extent, calculated as %RVD = [(A_rel,peak_ − A_rel,60 min_)/A_rel,peak_] x 100. Data are shown as mean ± SEM. Statistical significance was assessed using one-sample *t*-test against the theoretical value of 1 (A, *inset*) and one-way ANOVA followed by Tukey’s *post hoc* test (C). Individual data points are shown for each experimental condition. All experiments were performed in at least three independent preparations

We next investigated the contribution of K_Ca_ channels to swelling-activated K^+^ currents, through whole-cell perforated patch-clamp recordings. In the continuous presence of DCPIB to block VRAC-mediated Cl⁻ conductance, exposure to a 30% hypotonic solution elicited a prominent outward current with an approximately linear I-V relationship and a reversal potential near the K^+^ equilibrium potential ($\:{E}_{K}$≈ −80 mV), consistent with a K^+^-selective conductance (Fig. 2A). This current was almost completely abolished by 3 µM TRAM-34, whereas subsequent addition of 3 mM TEA had no detectable effect. Fractional inhibition at 0 mV was 97.3 ± 1.6% with TRAM-34 and only 2.7 ± 1.0% with TEA, confirming the absence of functional BK channel expression and establishing IK channels as the predominant mediators of swelling-activated K^+^ currents in GL261 cells.

To determine the functional contribution of IK channels to RVD, cell volume changes were monitored by phase-contrast microscopy. A 30% hypotonic challenge induced rapid cell swelling followed by near-complete volume recovery under control conditions (final recovery: 99.8 ± 3.3%; Fig. 2B, C), indicative of efficient RVD. Pharmacological inhibition of IK channels with 3 µM TRAM-34 markedly impaired RVD, reducing volume recovery to 22.9 ± 3.7%. In contrast, BK channel blockade with 3 mM TEA had no effect (93.5 ± 7.9%), consistent with the absence of functional BK channels in GL261 cells.

Collectively, these results demonstrate that IK channels are the sole functionally expressed K_Ca_ subtype in GL261 cells and are essential for mediating K^+^ efflux required for effective RVD.

### ADEVs downregulate IK channel expression in GL261 cells via miR-124

Given the essential role of IK channels in facilitating RVD, we next investigated whether ADEVs modulate their expression and functional activity. Based on our previous demonstration that ADEVs transfer miR-124 to glioma cells and suppress VRAC-mediated Cl⁻ conductance [9], we hypothesized that miR-124-enriched ADEVs additionally target IK channels, thereby affecting a second critical ionic pathway involved in volume regulation in GL261 cells.

**Fig. 3:**
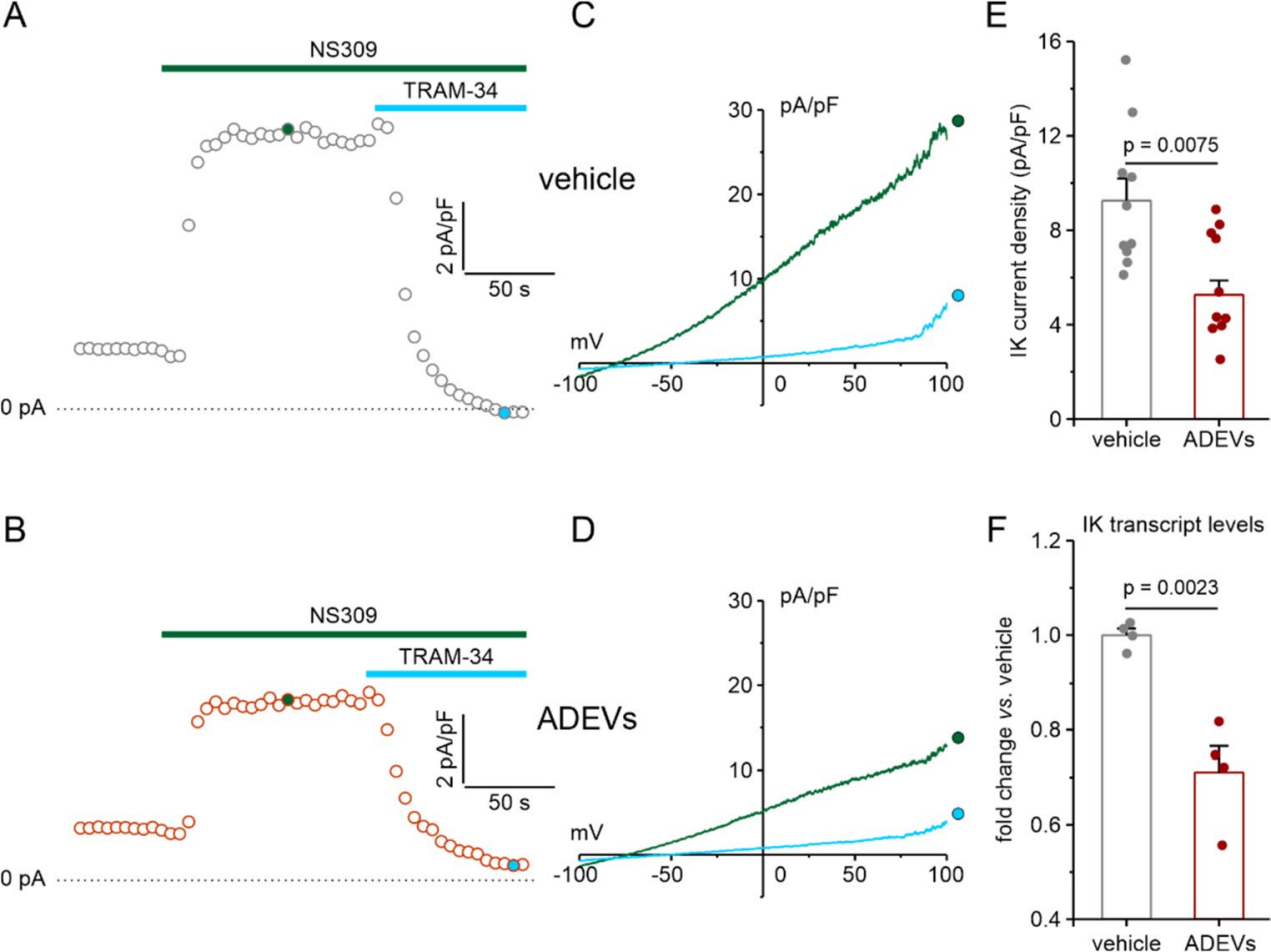
Assessment of IK channel function and transcript levels in GL261 cells following ADEVs treatment.** A**,** B**) Representative time courses of IK current density (pA/pF) at 0 mV in the presence of 400 nM intracellular Ca^2+^, under control conditions and during sequential application of 10 µM NS309 (dark green bar) and 3 µM TRAM-34 (cyan bar) in vehicle-treated (grey circles, A) and ADEV-treated (red circles, B) cells. **C**,** D**) Representative current traces evoked by voltage ramps from −100 to +100 mV (1 s duration; holding potential − 40 mV) following NS309 (dark green trace) and TRAM-34 (cyan trace) application in vehicle-treated (**C**) and ADEV-treated cells (**D**). **E**) Average IK current density (pA/pF) at 0 mV, calculated as the difference between NS309-activated and TRAM-34-inhibited currents, in vehicle-treated (grey bar, *n* = 10) and ADEV-treated (red bar, *n* = 10) cells. **F)** Average IK channel transcript levels quantified by RT-qPCR in vehicle-treated (grey bar; *n* = 4) and ADEV-treated (red bar; *n* = 4) cell cultures. Data are shown as mean ± SEM. Statistical significance was assessed using an unpaired two-sample *t* test. Individual data points are reported for each experimental condition. All experiments were performed in at least three independent preparations

Whole-cell dialyzed patch-clamp recordings performed under conditions isolating IK currents, as described in Fig. 1A, revealed a pronounced reduction in IK current amplitude following ADEV treatment (Fig. 3A-D). Quantitative analysis of TRAM-34-sensitive current density showed an approximately 50% decrease in current density in ADEV-treated cells (5.3 ± 0.6 pA/pF) compared with vehicle-treated controls (9.3 ± 0.9 pA/pF; Fig. 3E), indicating substantial functional downregulation of IK channels.

In agreement with these electrophysiological findings, RT-qPCR analysis demonstrated a ~ 30% reduction in *KCNN4* transcript levels in ADEV-treated cells relative to controls (Fig. 3F), supporting the conclusion that ADEVs downregulate IK channel expression at the mRNA level.

**Fig. 4:**
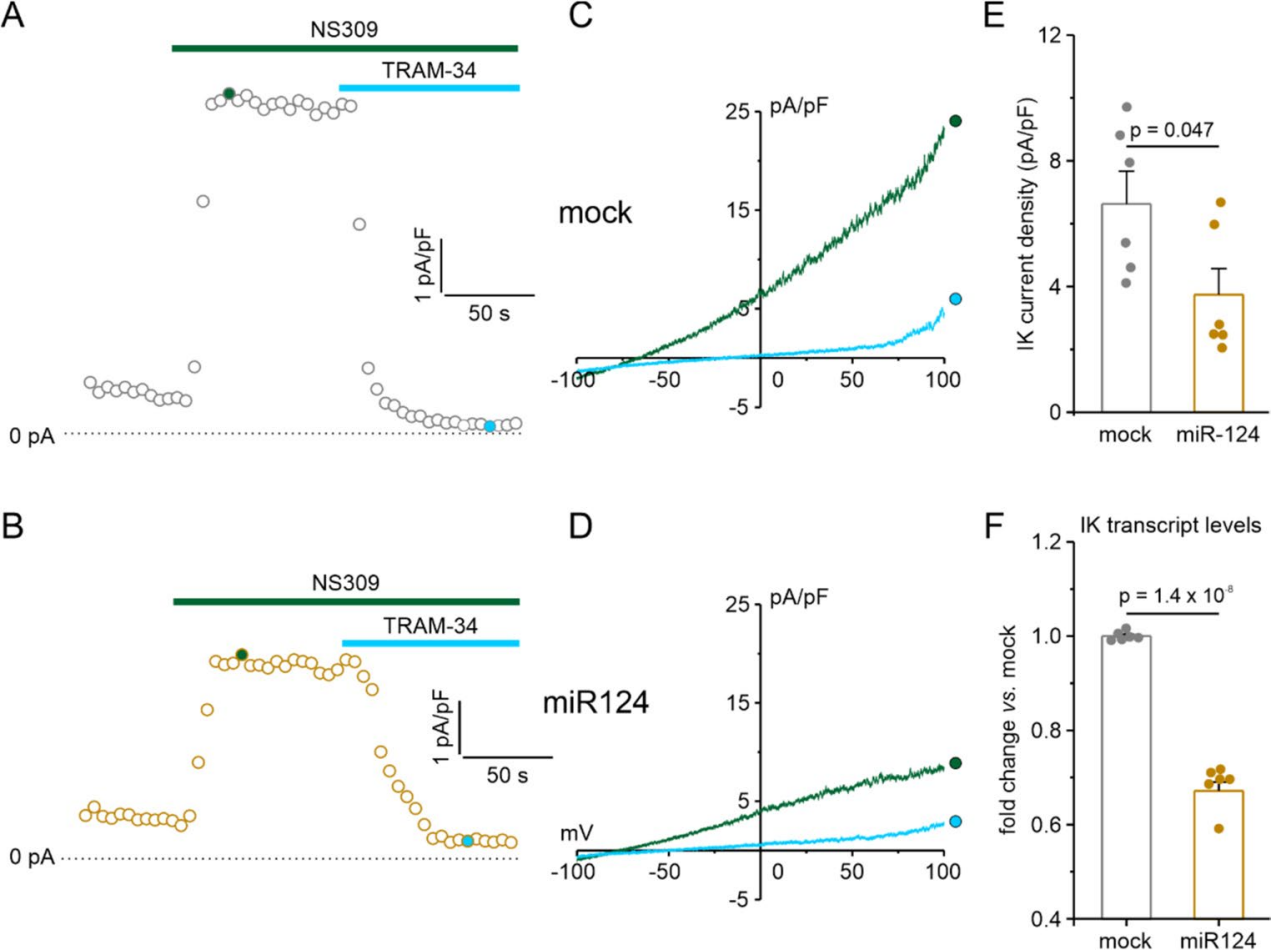
Assessment of IK channel function and transcript levels in GL261 cells following miR-124 transfection**.** **A**,** B***)* Representative time courses of IK current density (pA/pF) at 0 mV in the presence of 400 nM intracellular Ca^2+^, under control conditions and during sequential application of 10 µM NS309 (dark green bar) and 3 µM TRAM-34 (cyan bar) in mock-transfected (grey circles, A) and miR-124-transfected (yellow circles, B) cells. **C**,** D**) Representative current traces evoked by voltage ramps from −100 to +100 mV (1 s duration; holding potential − 40 mV) following NS309 (dark green trace) and TRAM-34 (cyan trace) application in mock-transfected (**C**) and miR-124-transfected cells (**D**). **E**) Average IK current density (pA/pF) at 0 mV, calculated as the difference between NS309-activated and TRAM-34-inhibited currents, in mock-transfected (grey bar, *n* = 6) and miR-124-transfected (red bar, *n* = 6) cells. **F)** Average IK channel transcript levels quantified by RT-qPCR in mock-transfected (grey bar; *n* = 6) and miR-124-transfected (red bar; *n* = 6) cell cultures. Data are shown as mean ± SEM. Statistical significance was assessed using an unpaired two-sample *t* test. Individual data points are reported for each experimental condition. All experiments were performed in at least three independent preparations

To assess whether the observed downregulation of IK channels was mediated by miR-124, GL261 cells were transfected with synthetic miR-124 or a mock control and subsequently analyzed for IK channel expression and function. In line with the effects observed following ADEV treatment, NS309-evoked IK currents were significantly reduced in miR-124-transfected cells whereas, at 0 mV the TRAM-34-sensitive current density decreased from 6.6 ± 1.0 pA/pF in mock-transfected cells to 3.8 ± 0.9 pA/pF in miR-124-transfected cells (Fig. 4A-E). Consistent with the electrophysiological data, RT-qPCR analysis revealed an ~ 35% reduction in *KCNN4* transcript levels in miR-124-transfected cells compared with controls (Fig. 4F), confirming a direct role for miR-124 in suppressing IK channel mRNA expression.

### ADEVs and miR-124 reduce ERK1/2 activation and IK channel expression by downregulating ER Ca^2+^-release channels

**Fig. 5:**
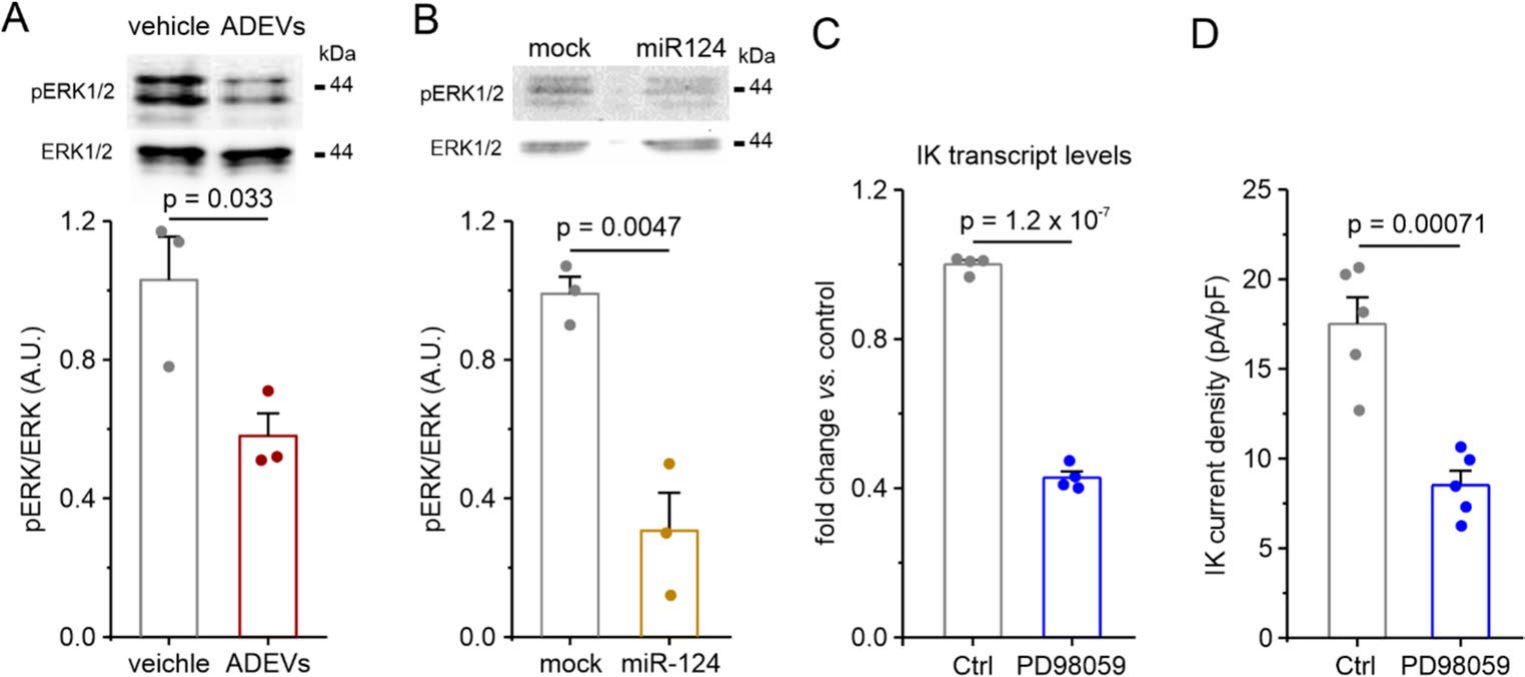
Impact of ADEVs treatment and miR-124 transfection on ERK1/2 phosphorylation and IK channel expression in GL261 cells.** A**,** B**) *Top*: Representative immunoblots showing total ERK1/2 and phosphorylated ERK1/2 (pERK1/2) in vehicle- versus ADEV-treated cells (A) and in mock- versus miR-124-transfected cells (B). Molecular weight markers (kDa) are indicated on the right. *Bottom*: Quantification of pERK1/2 normalized to total ERK1/2 in vehicle- (grey bar; *n* = 3) versus ADEV-treated (red bar; *n* = 3) cells, and in mock- (grey bar; *n* = 3) versus miR-124-transfected (yellow bar; *n* = 3) cells. **C)** Average IK transcript levels quantified by RT-qPCR in control-treated (grey bar, *n =* 4) and PD98059-treated (blue bar, *n =* 4) cell cultures. **D)** Average IK current density (pA/pF) at 0 mV, calculated as the difference between NS309-activated and TRAM-34-inhibited currents, in control-treated (grey bar, *n =* 5) and PD98059-treated (blue bar, *n =* 5) cells. Data are shown as mean ± SEM. Statistical significance was assessed using an unpaired two-sample *t*-test. Individual data points are reported for each experimental condition. All experiments were performed in at least three independent preparations

The ERK1/2 signaling cascade is a well-established regulator of IK channel transcription in GBM cells [28, 29]. Because *KCNN4* is not a predicted direct target of miR-124, we hypothesized that ADEVs downregulate IK channel expression and function indirectly through inhibition of ERK1/2 signaling. Western blot analysis demonstrated a significant reduction in ERK1/2 phosphorylation, a canonical indicator of pathway activation, in both ADEV-treated and miR-124-transfected GL261 cells, with miR-124 exerting a more pronounced inhibitory effect (Fig. 5A-B).

To verify the requirement of ERK1/2 activity for IK channel expression in the murine GL261 cell line, cells were treated with the MEK inhibitor PD98059. Pharmacological blockade of MEK/ERK signaling resulted in an ~ 60% decrease in *KCNN4* mRNA levels (Fig. 5C) and a marked decrease in NS309-evoked IK current density from 17.5 ± 1.5 to 7.9 ± 1.4 pA/pF (Fig. 5D). Together, these findings indicate that ERK1/2 activity is essential for maintaining IK channel transcription and support the conclusion that ADEV- and miR-124-mediated downregulation of IK channels occurs via suppression of ERK1/2 signaling rather than direct targeting of *KCNN4*.

**Fig. 6:**
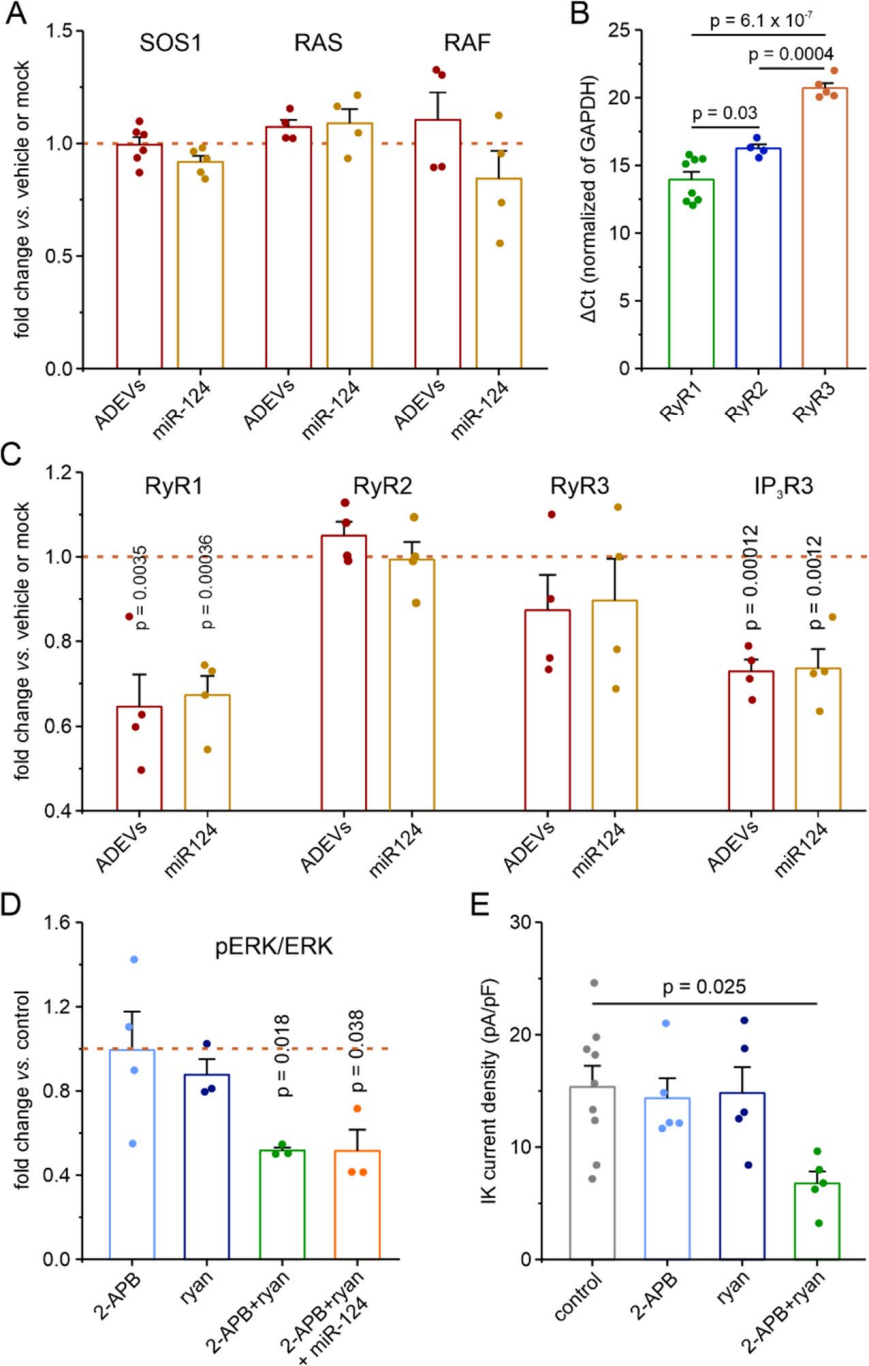
Effects of ADEVs and miR-124 on SOS1/RAS/RAF expression, ER Ca^2+^-release channel expression, ERK1/2 phosphorylation, and IK channel activity in GL261 cells. (**A**) *SOS1*, *RAS*, and *RAF* transcript levels quantified by RT-qPCR in ADEV-treated (red bars; *n* = 6 for *SOS1*, *n* = 4 for *RAS*, *n* = 4 for *RAF*) and miR-124-transfected (yellow bars; *n* = 5 for *SOS1*, *n* = 4 for *RAS* and *RAF*) cells. (**B**) Expression of RyR isoforms (RyR1; *n* = 8, RyR2; *n* = 4, and RyR3; *n* = 5) quantified by RT-qPCR and normalized to GAPDH as reference control. Values are reported as ΔCt (Ct_target_ − Ct_GAPDH_). (**C**) ER Ca^2+^-release channels *RyR1-3* and *IP*_*3*_*R3* transcript levels quantified by RT-qPCR in ADEV-treated (red bars; *n* = 4 for *RyR1*, *n* = 4 for *RyR2*, *n* = 4 for *RyR3*, and *n* = 4 for *IP*_*3*_*R3*) and in miR-124-transfected cells (yellow bars; *n* = 4 for *RyR1*, *n* = 4 for *RyR2*, *n* = 4 for *RyR3*, and *n* = 4 for *IP*_*3*_*R3*). (**D**) Densitometric analysis of phosphorylated ERK1/2 (pERK1/2) levels normalized to total ERK1/2 in cells treated with ER Ca^2+^-release channel inhibitors either individually (2-APB 100 µM, purple bar; *n* = 4 and ryanodine 50 µM, dark blue bar; *n* = 3) or in combination (2-APB + ryanodine; green bar; *n* = 3), and in cells co-treated with both inhibitors and miR-124 (2-APB + ryanodine + miR-124, orange bar; *n* = 3). **(E)** Average IK current density (pA/pF) at 0 mV, calculated as the difference between NS309-activated and TRAM-34-inhibited currents, in control cells (grey bar, *n =* 5), in cells treated with ER Ca^2+^-release channel inhibitors either individually (2-APB 100 µM, purple bar; *n* = 5 and ryanodine 50 µM, dark blue bar; *n* = 5) or in combination (2-APB + ryanodine; green bar; *n* = 5). Data are shown as mean ± SEM. In panels A, C, and D, values are expressed as fold of change relative to untreated control cells, which were normalized to 1 (dashed line) and not plotted as bars. Statistical significance in panels A, C, and D was assessed using an unpaired two-tailed *t*-test (each condition compared with its respective control), whereas panels B and E were analyzed using one-way ANOVA followed by Tukey’s *post hoc* test. Individual data points are reported for each experimental condition. All experiments were performed in at least three independent preparations

The canonical rat sarcoma/mitogen-activated protein kinase (RAS/MAPK) pathway is regulated by multiple upstream components, including son of sevenless 1 (SOS1) and rapidly accelerated fibrosarcoma (RAF), which are central mediators of proliferative signaling [30]. Since SOS1 and RAS have been identified as miR-124 targets in glioma cells [31, 32], and bioinformatic analyses (miRDB) predicted RAF as a potential miR-124 targets, we examined whether ADEVs or miR-124 alter the expression of these components in GL261 cells. However, RT-qPCR analysis revealed no significant changes in SOS1, RAS, or RAF transcript levels following either ADEV treatment or miR-124 transfection (Fig. 6A), indicating that miR-124 does not suppress ERK signaling via direct repression of canonical RAS/MAPK components.

Substantial evidence indicates that intracellular Ca^2+^ dynamics are well-established regulators of ERK activation. For example, Ca^2+^ signaling enhances HIF-1-dependent ERK activation under hypoxic conditions [33], depolarization-induced Ca^2+^ influx robustly activates ERK in hippocampal neurons [34, 35], and mechanical stimulation activates ERK via Piezo1-mediated Ca^2+^ entry [36]. A major source of intracellular Ca^2+^ signals is the ER, where Ca^2+^ release is primarily mediated by two families of intracellular channels: IP_3_Rs and RyRs. Notably, miR-124 has been reported to target ER Ca^2+^ release channels, including IP_3_R3 and all three RyR isoforms (RyR1-3), thereby attenuating receptor-mediated Ca^2+^ mobilization while preserving basal Ca^2+^ levels [37]. These observations prompted us to investigate whether ADEV-delivered miR-124 attenuates ERK1/2 activation by disrupting ER-mediated Ca^2+^ release in GL261 cells.

While IP_3_R3 has been identified as the predominant IP_3_R isoform in glioma cells and plays a key role in tumor migration and invasion [38], the expression pattern of RyR isoforms in this context remains poorly defined, although RyR3 has been identified as a prognostic marker in GBM [39]. We therefore first characterized the expression profile of RyR isoforms in GL261 cells. RT-qPCR analysis demonstrated that RyR1 is the predominant isoform, expressed at markedly higher levels than RyR2 and RyR3 (Fig. 6B). Importantly, miR-124 transfection or ADEV treatment significantly reduced RyR1 expression, whereas RyR2 and RyR3 levels remained essentially unchanged (Fig. 6B). Conversely, IP_3_R3 transcripts were similarly downregulated under both conditions (Fig. 6C). Together, these data identify RyR1 and IP_3_R3 as the principal ER Ca^2+^ release channels modulated by miR-124 in GL261 cells.

We therefore hypothesized that impaired ER Ca^2+^ release through RyR1 or IP_3_R3 underlies the reduction of ERK1/2 phosphorylation and the subsequent decrease in IK channel expression. To test this hypothesis, ER Ca^2+^ release was pharmacologically inhibited using saturating concentrations of 2-APB (100 µM) and ryanodine (50 µM), selective antagonists of IP_3_Rs and RyRs, respectively.

To determine which of the two Ca^2+^ release pathways contributes to ERK1/2 phosphorylation, measurements were performed in the presence of each blocker alone or in combination. While individual inhibition of IP_3_R3 or RyR1 did not significantly alter ERK1/2 phosphorylation, it was markedly reduced upon dual blockade, phenocopying the effects of ADEVs and miR-124. Notably, co-application of miR-124 with the two blockers did not produce further suppression, indicating that its effect on ERK1/2 signaling is largely mediated by downregulation of ER Ca^2+^ release channels (Fig. 6D). We next assessed the impact of impaired ER Ca^2+^ signaling on IK channel functional expression. Consistent with the ERK1/2 phosphorylation data, functional experiments revealed that individual inhibition of IP_3_R3 or RyR1 did not significantly decrease NS309-activated, TRAM-34-sensitive IK current density (14.3 ± 1.8 pA/pF and 14.8 ± 2.3 pA/pF in the presence of 2-APB and ryanodine, respectively) compared with control cells (15.4 ± 1.9 pA/pF). In contrast, dual inhibition produced a significant reduction in NS309-elicited IK current density (6.8 ± 1.1 pA/pF), closely matching the decrease observed following miR-124 treatment and substantially lower than control values (Fig. 6E).

Collectively, these findings support a model in which ADEV-delivered miR-124 suppresses IK channel expression not by directly targeting canonical RAS/MAPK components but through coordinated transcriptional downregulation of ER Ca^2+^-release channels, predominantly RyR1 and IP_3_R3. The resulting attenuation of ER Ca^2+^ signaling limits ERK1/2 activation and ultimately reduces IK channel expression and function.

### miR-124 and ADEVs impair FBS-induced IK channel activation and cell migration by disrupting ER Ca^2+^-dependent signaling

In GBM cells, IK channel activation is primarily driven by Ca^2+^ release from ER stores. Given that ADEVs and miR-124 downregulate ER Ca^2+^ release channels, we hypothesized that these interventions not only reduce IK channel expression but also compromise the activation of residual channels in response to physiologically relevant stimuli that depend on ER-derived Ca^2+^.

**Fig. 7:**
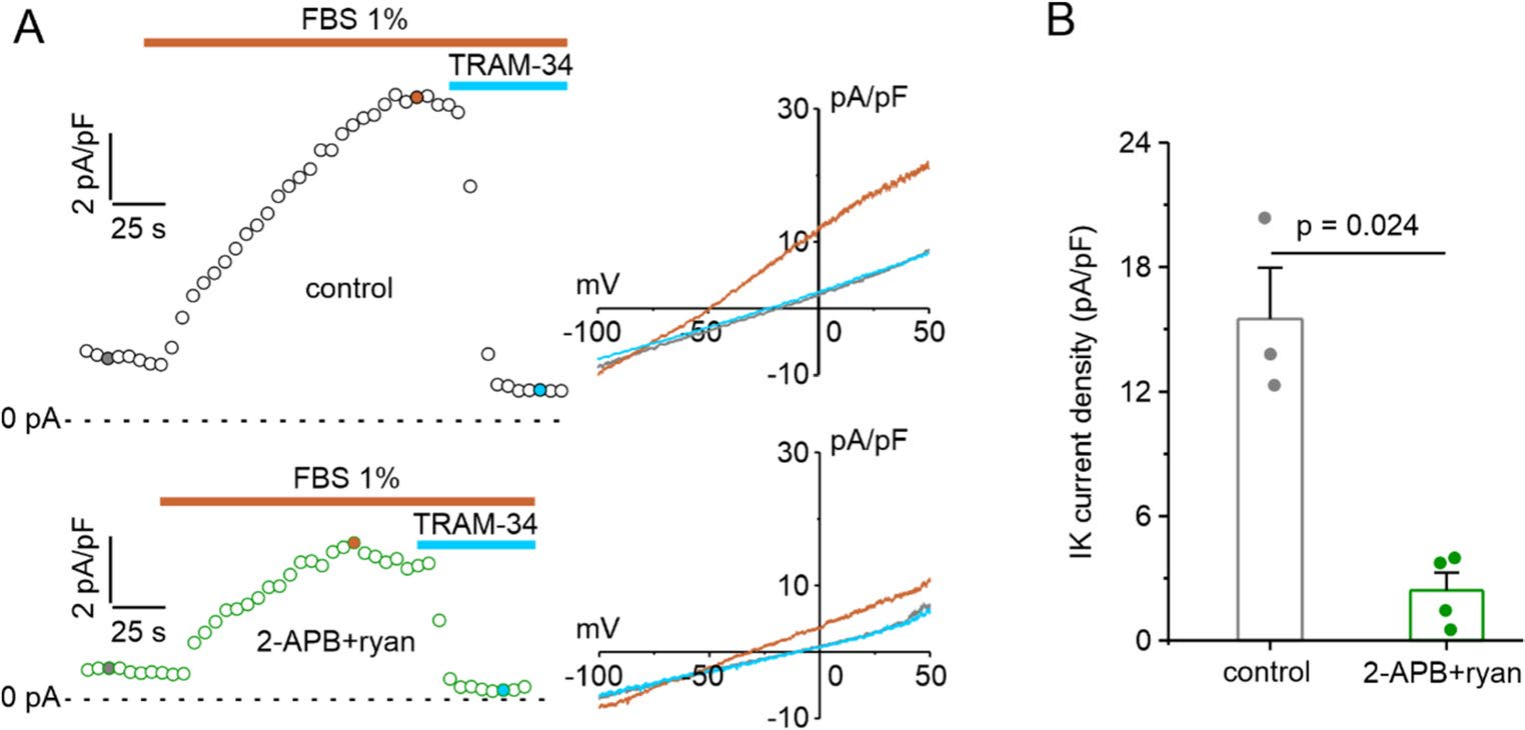
FBS-induced IK current activation in the presence or absence of ER Ca^2+^-release channel inhibitors. (**A**) *Left*: Representative time courses of IK current density (pA/pF) at 0 mV in response to 1% FBS (dark orange bar) and subsequent application of 3 µM TRAM-34 (cyan bar), in untreated control cells (top, grey circles) and in cells pre-treated with the combination of ER Ca^2+^-release channel inhibitors 2-APB (100 µM) and ryanodine (50 µM) (bottom, green circles). *Right*: Representative current traces evoked by voltage ramps from −100 to +100 mV (holding potential − 40 mV) corresponding to the symbols in the time courses having the same color. (**B**) Average FBS-induced IK current density (pA/pF) at 0 mV, calculated as the difference between the current activated by FBS and the residual current after TRAM-34 application, in untreated control cells (grey bar; *n* = 3) and in cells treated with 2-APB + ryanodine (green bar; *n* = 4). Data are shown as mean ± SEM. Statistical significance was assessed using an unpaired two-sample t-test. Individual data points are reported for each experimental condition. All experiments were performed in at least three independent preparations

To test this hypothesis, we examined IK channel activation in response to FBS, a well-established stimulus that triggers PLC-dependent ER Ca^2+^ mobilization and subsequent IK channel activation [40–42]. Experiments were performed using whole-cell perforated patch-clamp recordings to preserve the integrity of intracellular Ca^2+^ signaling pathways during receptor-mediated stimulation. We first confirmed that FBS activates IK channels in GL261 cells via ER Ca^2+^ release (Fig. 7A, B). Acute exposure to 1% FBS elicited a robust, voltage-independent outward current at 0 mV that was fully blocked by TRAM-34 under control conditions (Fig. 7A). In contrast, combined pharmacological inhibition of IP_3_Rs and RyRs with 100 µM 2-APB and 50 µM ryanodine markedly attenuated this response, reducing the FBS-evoked IK current density from 15.5 ± 2.5 pA/pF in control cells to 2.4 ± 0.9 pA/pF (Fig. 7B).

These results demonstrate that ER-derived Ca^2+^ release is essential for FBS-induced IK channel activation in GL261 cells and provide a mechanistic basis for the effects induced by ADEVs and miR-124 treatment. By downregulating IP_3_Rs and RyRs, ADEVs and miR-124 disrupt Ca^2+^-dependent IK signaling, thereby impairing IK-mediated downstream processes, including three-dimensional glioma cell migration.

We next examined whether the miR-124-dependent reduction of ER Ca^2+^ release channels functionally impairs FBS-induced IK channel activation. IK currents were therefore measured in control cells and in cells exposed to ADEVs or transfected with miR-124. FBS-induced IK channel activation was quantified as the fraction of the TRAM-34-sensitive current elicited by FBS relative to the maximal IK current obtained by co-application of FBS with the selective IK channel activator NS309. This normalization accounted for the reduced IK channel expression caused by ADEVs and miR-124 (Figs. 3 and 4) and allowed selective assessment of ER Ca²⁺-dependent channel activation.

**Fig. 8:**
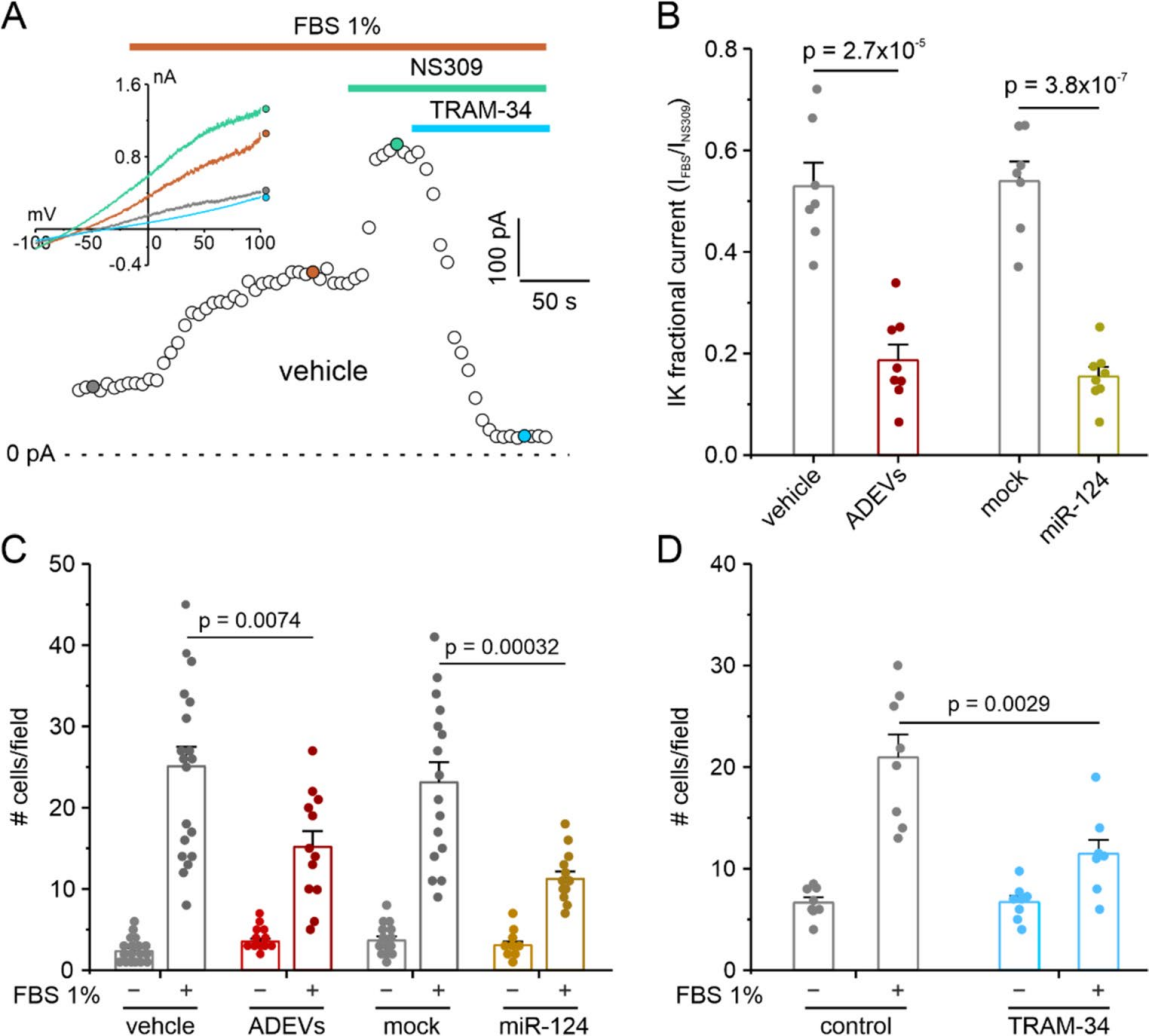
Effects of ADEVs treatmen and miR-124 transfection on FBS-induced IK activation and three-dimensional migration in GL261 cells. (**A**) Representative time course of IK current (pA) at 0 mV in response to 1% FBS (dark orange bar), followed by 10 µM NS309 (green bar) and 3 µM TRAM-34 (cyan bar) in a vehicle-treated control cell. *Inset*: Representative current traces evoked with voltage ramps (−100 to +100 mV; holding potential − 40 mV) corresponding to the symbols of the time course having the same color. (**B**) Fractional IK current activation, calculated as the TRAM-34-sensitive current elicited by 1% FBS (I_FBS_) normalized to the NS309-activated current (I_NS309_), in vehicle-treated control (grey bar; *n* = 7), ADEVs-treated (red bar; *n* = 8), mock-transfected control (grey bar; *n* = 8), and miR-124-transfected (yellow bar; *n* = 8) cells. **C, D**) Transwell migration assays showing the number of GL261 cells migrating under different conditions. Cells were assessed in the absence (–) or presence (+) of 1% FBS after vehicle treatment (grey bars, *n* = 20), ADEV treatment (red bars, *n* = 20), mock transfection (grey bars, *n* = 20), or miR-124 transfection (yellow bars, *n* = 20) (C). The effect of IK channel inhibition was evaluated by treating control cells with 3 µM TRAM-34 (cyan bars, *n* = 6) under identical FBS stimulation (D). Data are shown as mean ± SEM. Statistical significance was assessed using an unpaired two-sample *t*-test. Individual data points are reported for each experimental condition. All experiments were performed in at least three independent preparations

Both ADEV-treated and miR-124-transfected cells exhibited more than a 50% reduction in the fraction of FBS-activated IK current compared with vehicle-treated or mock-transfected controls (Fig. 8A, B), indicating a pronounced impairment of FBS-evoked IK channel activation when ER Ca^2+^-release channels are downregulated. These findings underscore the requirement for ER Ca^2+^ mobilization as an essential upstream step in FBS-mediated IK channel activation and demonstrate that ADEVs and their cargo miR-124 attenuate this signaling axis via transcriptional repression of IP_3_Rs and RyRs, thereby weakening the coupling between extracellular stimulation and IK channel opening.

Given that IK channel activity promotes GBM cell migration and invasion [9, 40, 43], we next evaluated whether ADEV- and miR-124-mediated suppression of IK channels translates into reduced GL261 cell motility. Using a three-dimensional transwell migration assay with FBS as the chemoattractant, both ADEV-treated and miR-124-transfected cells exhibited a significant reduction in FBS-driven migration compared with their respective controls, with an approximately twofold decrease in the number of migrated cells (Fig. 8C). Notably, pharmacological inhibition of IK channels with 3 µM TRAM-34 produced a comparable impairment in migration (Fig. 8D).

Collectively, these results demonstrate that the anti-migratory effects of ADEVs and miR-124 are mediated, at least in part, by suppression of IK channel expression and concomitant attenuation of ER Ca^2+^-dependent IK channel activation.

### ADEVs impair RVD through dual inhibition of VRAC and IK channels

The ADEV-induced downregulation of IK channels is expected to substantially contribute to the ~50% reduction in RVD previously observed in GL261 cells, which was attributed in part to a ~ 40% decrease in VRAC expression and function [9]. Given the magnitude of the RVD impairment, inhibition of VRAC alone is unlikely to fully account for the phenotype. We therefore hypothesized that concurrent suppression of both VRAC and IK channels is required to reproduce the RVD impairment observed with ADEVs.

To test this hypothesis, RVD was analyzed by live-cell imaging under conditions of controlled, partial inhibition of VRAC and IK channels. DCPIB and TRAM-34 were applied at concentrations selected to produce ~40% inhibition of each channel, thereby approximating the degree of functional suppression observed following ADEV treatment.

**Fig. 9:**
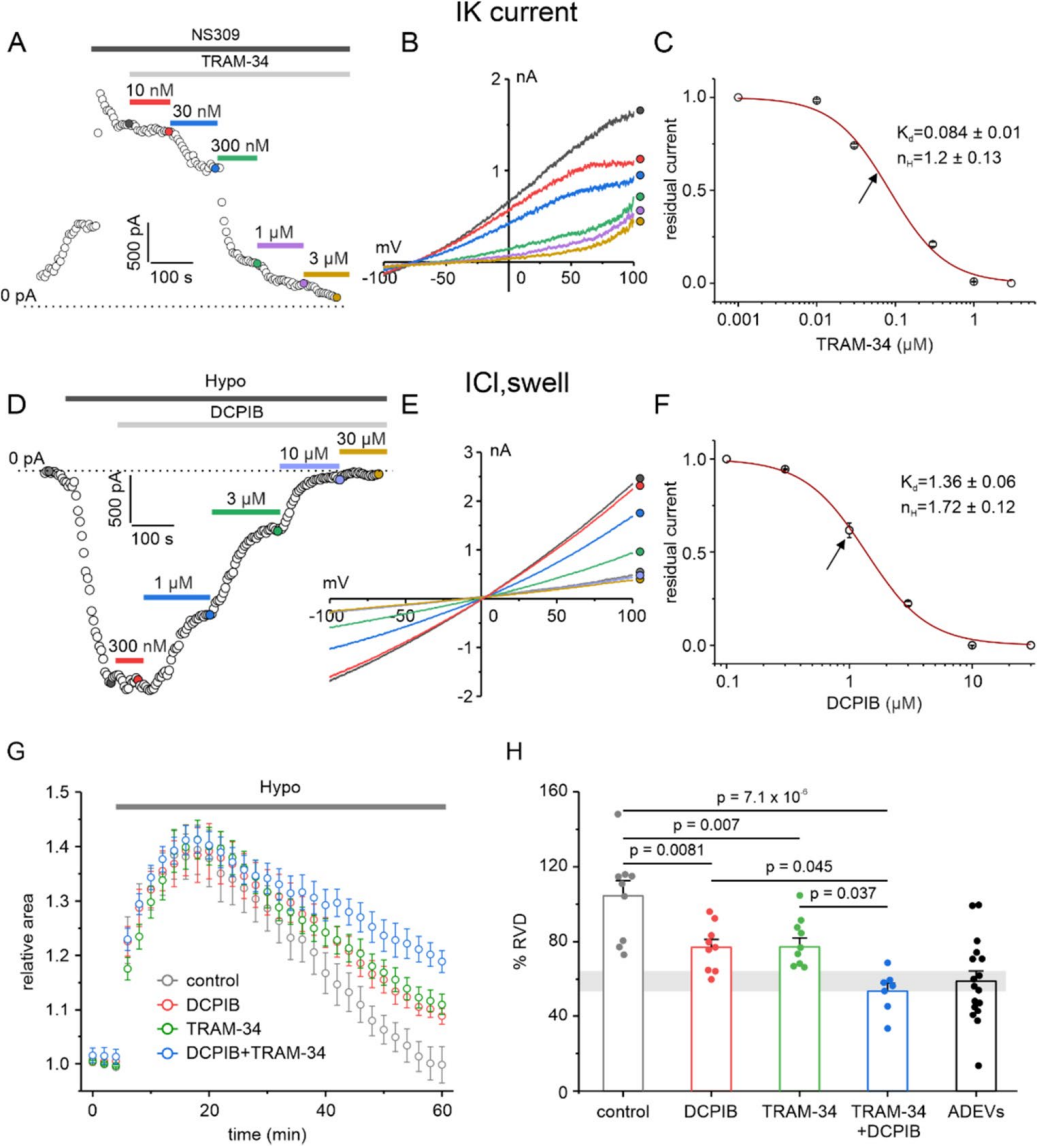
Dose-dependent inhibition of IK and VRAC currents and effects on RVD in GL261 cells.** A-C**) TRAM-34 dose-response for IK inhibition. (A) Representative time course of IK current (pA) at 0 mV following activation with 400 nM intracellular Ca^2+^ and 10 µM NS309 (grey bar), with sequential application of TRAM-34 at 10 nM (red), 30 nM (blue), 300 nM (green), 1 µM (purple), and 3 µM (yellow). (**B**) Representative current traces evoked by voltage ramps from −100 to +100 mV (holding potential −40 mV) corresponding to the time course. (**C**) Fractional residual IK current as a function of TRAM-34 concentration. Continuous line represents the best fit to a Hill equation (K_d_ = 0.084 µM and n_H_ = 1.2). The black arrow indicates 50 nM TRAM-34, yielding ~40% inhibition, used in RVD assays (G-H). **D-F**) DCPIB dose-response for VRAC inhibition. (**D**) Representative time course of VRAC-mediated I_Cl,swell_ (pA) at −80 mV during exposure to 30% extracellular hypotonic solution (Hypo, grey bar), followed by sequential addition of DCPIB at 300 nM (red trace), 1 µM (blue bar), 3 µM (green bar), 10 µM (purple bar), and 30 µM (yellow bar). (**E**) Representative current traces evoked by voltage ramps from −100 to +100 mV (holding potential −40 mV) corresponding to the symbols of the time course having the same color. (**F**) Fractional residual I_Cl, swell_ as a function of DCPIB concentration. Continuous line represents the best fit to a Hill equation (K_d_ = 1.34 µM and n_H_ = 1.72). The black arrow indicates 1 µM DCPIB concentration, yielding ~40% inhibition, used in RVD assays (G, H). (**G**) Time course of RVD measured as changes in relative cell projected area during exposure to 30% hypotonic solution (Hypo, grey bar) in control cells (grey circles, *n* = 9), partial VRAC inhibition (DCPIB 1 µM; red circles, *n* = 9), partial IK channels inhibition (TRAM-34 50 nM; green circles, *n* = 9), or combined inhibition (DCPIB + TRAM-34; blue circles, *n* = 7). (**H**) Average RVD extent, calculated as %RVD (see Fig. 2) for all experimental conditions. Data from ADEVs-treated cells [9] are included for comparison. Data are shown as mean ± SEM. Statistical significance was assessed using one-way ANOVA followed by Tukey’s *post hoc* test. Individual data points are reported for each experimental condition. All experiments were performed in at least three independent preparations

Dose-response relationships were first established using whole-cell patch-clamp recordings (Fig. 9A-F). IK currents were measured at 0 mV using 1-s voltage ramps from −100 to +100 mV (holding potential −40 mV) in the presence of 400 nM intracellular Ca^2+^ and NS309. Sequential application of TRAM-34 (10 nM–3 µM) yielded an apparent K_d_ of ~84 nM and a Hill coefficient of 1.2, with 50 nM TRAM-34 producing ~40% inhibition (Fig. 9A-C).

VRAC currents (I_Cl, swell_) were elicited under 30% hypotonic conditions and quantified at − 80 mV using identical ramp protocols. Increasing concentrations of DCPIB (100 nM-30 µM) were applied sequentially, and residual currents were normalized to peak I_Cl, swell_. Fitting with the Hill relationship yielded an apparent K_d_ of ~ 1.4 µM and a Hill coefficient of ~ 1.72 (Fig. 9D-F); accordingly, 1.0 µM DCPIB was selected to achieve ~ 40% VRAC inhibition.

We next assessed the impact of partial channel inhibition on RVD. Upon exposure to a 30% hypotonic solution, partial inhibition of either VRAC or IK channels alone caused only modest impairment of volume recovery (%RVD = 77.0% and 77.3%, respectively), whereas control cells fully restored their volume (%RVD = 104.5%; Fig. 9G). In striking contrast, simultaneous partial inhibition of both channels produced a pronounced defect in volume recovery (%RVD = 53.5%; Fig. 9H), closely recapitulating the phenotype observed in ADEV-treated cells (%RVD = 59%; [9]).

Together, these results demonstrate that efficient RVD in GL261 cells requires the coordinated activity of both VRAC and IK channels, and that partial inhibition of both channels is necessary to reproduce the RVD deficit induced by ADEVs. More broadly, the data provide compelling evidence that miR-124 delivered via ADEVs disrupts glioma cell volume homeostasis through simultaneous suppression of VRAC and IK channels, identifying the IK channel, alongside VRAC, as a critical functional target through which ADEVs modulate glioma cell functions.

## Discussion

This study uncovers a previously unrecognized mechanism by which astrocytes regulate glioma cell volume homeostasis, a critical determinant of tumor invasiveness and progression. We demonstrate that ADEVs, through delivery of miR-124, selectively downregulate IK channel expression and function in GL261 glioma cells via a Ca^2+^-dependent signaling cascade. Specifically, miR-124 reduces ER Ca^2+^ signaling by targeting IP_3_R3 and RyR1, resulting in attenuated ERK1/2 phosphorylation and diminished transcription and stimulus-dependent activation of IK channels. This coordinated suppression impairs both RVD and three-dimensional migration, key processes underlying GBM progression. These findings extend our previous work showing that ADEV-delivered miR-124 suppresses VRAC expression and activity in GL261 cells [9], highlighting that astrocytes modulate glioma physiology through multiple, functionally interconnected ion channel pathways. Although the present study was conducted in vitro, its physiological relevance is supported by prior in vivo evidence demonstrating that ADEVs and miR-124 reduce GL261 tumor growth by limiting proliferation and invasiveness [9]. Nevertheless, future studies using more physiologically relevant experimental systems, such as glioma-bearing brain slices or in vivo models, will be important to further validate the ER Ca^2+^ release-ERK-IK channel signaling pathway described here within the native tumor microenvironment. Collectively, these results establish extracellular vesicle-mediated microRNA transfer as a potent mechanism by which astrocytes constrain GBM aggressiveness.

### miR-124 suppresses IK channels via a non-canonical ER Ca^2+^-dependent pathway

*KCNN4* is not a predicted target of miR-124, unlike the VRAC subunit *LRRC8C* [9], indicating that IK channel suppression likely occurs through miR-124-regulated signaling intermediates. Both ADEV treatment and miR-124 transfection reduce ERK1/2 phosphorylation, a regulator of IK channel transcription in GBM cells [28, 29]. MEK inhibition with PD98059 recapitulates these effects, decreasing *KCNN4* transcript levels and IK current density. Notably, transcripts encoding canonical growth factor receptor signaling components upstream of ERK1/2, including SOS1, RAS, and RAF, remain unchanged by ADEV treatment or miR-124 overexpression, indicating that they are not involved in miR-124-dependent ERK1/2 inactivation in GL261 cells. This contrasts with previous studies in human GBM models, where miR-124 directly targets upstream MAPK regulators (SOS1, R-RAS, N-RAS), suppressing downstream ERK signaling [31, 32]. While the reason for this discrepancy is unknown, it raises the possibility that the signaling mechanisms described here are restricted to murine glioma cell lines or are present only in a subset of human GBMs.

We identify a noncanonical ER Ca^2+^-dependent pathway whereby miR-124 delivered by ADEVs reduces transcripts encoding IP_3_Rs and RyRs, the principal ER Ca^2+^ release channels. Direct targeting of these channels by miR-124 has been reported in human neuroblastoma cells [37], and our findings demonstrate functional relevance in murine glioma cells. Combined inhibition of both IP_3_Rs and RyRs closely phenocopies the effects of miR-124 on ERK1/2 phosphorylation and IK channel activity, whereas inhibition of either channel alone is insufficient. Co-application of miR-124 with ER Ca^2+^ release inhibitors does not produce additive suppression, supporting the concept that miR-124 acts upstream by limiting ER Ca^2+^ mobilization, consistent with Paschou et al. [37].

Isoform-specific analysis reveals that RyR1 is the predominant RyR isoform in GL261 cells and the only isoform significantly downregulated by miR-124, even if RyR3, but not RyR1, has been identified as a prognostic marker in human GBM [39]. IP_3_R3, identified as the predominant IP_3_R isoform in glioma cells and implicated in glioma invasiveness [38], is also reduced by miR-124. These results may suggest that in GL261 cells, RyR1 and IP_3_R3 form a functional ER Ca^2+^ signaling axis that controls ERK1/2 activation and IK channel functional expression, thereby linking miRNA-mediated regulation to ion channel-dependent signalling dynamics in glioma cells. Our observation that only RyR1, but not RyR2 or RyR3, is modulated by miR-124 appears to contrast with the findings of Paschou and colleagues, who reported that miR-124 downregulates all three RyR isoforms in the SK-N-SH neuroblastoma cell line [37]. This divergence is likely attributable to the distinct neural lineage and cellular identity of neuroblastoma and GBM. Importantly, because these findings were obtained in the GL261 murine glioma model, further studies in additional murine and human glioma systems will be required to determine whether the selective regulation of RyR1 and the ER Ca^2+^-ERK-IK channel signaling axis described here represent a general mechanism in glioma biology or reflect model-specific features.

### Astrocyte versus GBM differential effects of miR-124

Although astrocytes express high endogenous levels of miR-124, they maintain effective volume control. This resilience likely reflects differences in K^+^ channel expression profiles between astrocytes and GBM cells. Astrocytes express low IK channel levels, largely confined to perivascular endfeet [44], and rely predominantly on K_ir_ channels for volume homeostasis [45, 46]. In contrast, GBM cells exhibit high IK channel expression that is functionally coupled to intracellular Ca^2+^ signaling, making them more susceptible to miR-124-mediated disruption of volume regulation. This differential sensitivity explains the tumor-selective impairment of RVD and highlights a potential therapeutic window for selectively targeting IK channel-dependent volume regulation in glioma cells.

### Complementary Ca^2+^ regulatory mechanisms: Piezo1 and ER Ca^2+^ release

While the present study identifies an ER Ca^2+^-dependent miR-124/IK regulatory axis, previous works have demonstrated that IK channel activity, RVD, and GBM invasiveness can also be regulated by the mechanosensitive Ca^2+^ channel Piezo1 [8, 25, 47–49]. These two Ca^2+^ sources likely represent complementary regulatory mechanisms rather than mutually exclusive pathways. Piezo1 mediates localized Ca^2+^ influx in response to mechanical stimuli, whereas ER Ca^2+^ release through IP_3_Rs and RyRs is primarily triggered by ligand- or receptor-mediated signaling. Given the high Ca^2+^ sensitivity of IK channels (EC50 ≈ 200 nM), both mechanisms can converge to promote channel activation, with Piezo1 contributing primarily during mechanotransduction and ER Ca^2+^ release during ligand-driven stimulation. In agreement with this model, we show that miR-124 delivered by ADEVs attenuates IK channel activation in response to FBS, a pro-migratory stimulus known to induce ER Ca^2+^ release [40]. Pharmacological inhibition of IP_3_Rs and RyRs reproduces this effect, indicating that intact ER Ca^2+^ signaling is required for stimulus-dependent IK activation. As a consequence, GL261 cells exhibit both reduced IK channel availability and diminished responsiveness to physiological stimuli, resulting in impaired three-dimensional migration. These observations are consistent with previous studies linking IK channel activity to glioma cell motility and invasiveness [12, 43, 50–52] and identify disruption of ER Ca^2+^ signaling as a mechanism through which ADEV-delivered miR-124 limits IK channel-dependent invasive behavior. Future studies in murine and human GBM models will be required to define the relative contributions of ER-derived and mechanosensitive Ca^2+^ signals to IK channel regulation and to determine whether ADEV-delivered miR-124 also modulates Piezo1-dependent pathways.

### Cooperative regulation of RVD by IK and VRAC channels

RVD is a tightly coordinated homeostatic process requiring coupled K^+^ and Cl⁻ efflux. Our findings refine this framework by demonstrating that IK and VRAC channels act cooperatively to ensure efficient volume recovery in GL261 glioma cells. While ADEVs or miR-124 reduce VRAC function by ~40%, this reduction alone is insufficient to account for the ~50% impairment of RVD previously reported [9]. Partial inhibition of either VRAC or IK channels alone, to an extent comparable to that induced by ADEVs, resulted in only moderate attenuation of volume recovery. In contrast, concurrent partial inhibition of both channels fully recapitulated the RVD deficit observed in ADEV-treated cells, underscoring the synergistic contribution of K^+^ and Cl⁻ fluxes to effective volume regulation.

### Model-specific divergence in K_Ca_ channel expression

The GL261 phenotype contrasts with human GBM models such as U87-MG, where both IK and BK channels are functionally expressed and cooperate to regulate RVD and migration [7, 13, 25, 49]. In contrast, electrophysiological and molecular analyses indicate that the IK channel is the only clearly functional K_Ca_ channel expressed in GL261 cells, where it plays a central role in volume regulation and FBS-induced three-dimensional migration. An outwardly rectifying BK-like current can occasionally be detected; however, this current is insensitive to TEA and paxilline and is not affected by miR-124 (data not shown). Therefore, its molecular identity was not investigated in the present study and remains undefined. Importantly, this current does not participate in the ER Ca^2+^-IK channel regulatory pathway described here, which represents the primary focus of our work. The predominance of IK channel activity in GL261 cells underscores model-specific differences and highlights the need for caution when extrapolating findings from murine to human glioma systems. Analysis of clinical datasets further reveals marked heterogeneity in K_Ca_ channel expression across human gliomas, supporting IK channels as a more consistent therapeutic target. BK channel upregulation occurs in approximately 10% of tumors and does not correlate with patient outcome, whereas IK channels are upregulated in about 32% of gliomas and are associated with poorer survival [12]. These observations suggest that the lack of BK channel expression in GL261 cells may reflect tumor heterogeneity rather than a strictly species-specific feature.

Despite these differences, the ER Ca^2+^/miR-124/IK channel regulatory pathway identified in GL261 cells is likely relevant to human GBM models expressing both IK and BK channels. In U87-MG cells, both channels contribute to volume regulation; however, inhibition of either channel alone impairs RVD to a similar extent as simultaneous blockade of both channels [7, 25]. This observation raises the possibility that selective suppression of IK channels by ADEV-delivered miR-124 could be sufficient to disrupt RVD and migration even in GBM cells that retain BK channel activity. Although consistent with the established role of both IK and BK channels in U87-MG cells, this hypothesis will require direct experimental validation across additional GBM models to determine its broader translational relevance.

### Limitations

Several limitations should be noted. First, all experiments were conducted in a single murine glioma model (GL261 cells), which may limit generalizability to human GBM. GL261 cells exhibit distinct K_Ca_ and RyR/IP_3_R expression profiles and species-specific regulation of canonical growth factor pathways. However, using a murine model allowed the isolation of sufficient quantities of ADEVs from astrocytes, which would be challenging in human systems. Second, the precise interplay between RyR1 and IP_3_R3 in sustaining intracellular Ca^2+^ dynamics required for ERK1/2 phosphorylation remains incompletely defined, as does the contribution of microdomain-restricted Ca^2+^ signals or oscillations, which are critical for efficient MAPK/ERK activation [53]. Third, ER Ca^2+^ release and intracellular Ca^2+^ transients were not directly measured; instead, IK channel activity, highly sensitive to Ca^2+^ (K_d_ ≈ 180 nM in human GBM cells) [28], served as an indirect but reliable functional readout of Ca^2+^-dependent signaling. Finally, all experiments were performed in vitro, which may not fully capture the complexity of the tumor microenvironment. Nevertheless, previous in vivo studies demonstrate that ADEVs and miR-124 suppress GL261 tumor growth and invasion [9], supporting the physiological relevance of our findings. Future work employing additional murine and human glioma models, combined with high-resolution Ca^2+^ imaging and selective manipulation of RyR1 and IP_3_R3, will be essential to validate and extend the ER Ca^2+^/IK regulatory axis described here.

## Conclusions and therapeutic implications

In summary, we identify a previously unrecognized Ca^2+^-dependent mechanism through which ADEV-delivered miR-124 disrupts glioma cell volume homeostasis and migration. By downregulating ER Ca^2+^ release channels, miR-124 attenuates intracellular Ca^2+^ signaling, suppresses ERK1/2 activation, and impairs both the expression and stimulus-dependent activation of IK channels. This coordinated disruption compromises RVD and three-dimensional migration, processes essential for GBM progression.

These results highlight IK channels and their upstream Ca^2+^-regulatory machinery as promising therapeutic targets. Strategies aimed at modulating ER Ca^2+^ dynamics, enhancing miR-124 delivery, or engineering ADEVs to selectively regulate ion channel-dependent pathways may provide innovative approaches to limit GBM invasiveness and progression. Future studies will be required to assess whether manipulation of these pathways can effectively reduce tumor aggressiveness and improve therapeutic outcomes in both murine and human GBM models.

## Data Availability

The data supporting the findings of this study are available from the corresponding author upon reasonable request.

## Abbreviations

ADEVs: astrocyte-derived extracellular vesicles
BK: large-conductance Ca^2+^-activated K^+^ channels
ER: endoplasmic reticulum
ERK: extracellular signal-regulated kinase
EVs: extracellular vesicles; FBS, fetal bovine serum
GBM: glioblastoma
I_Cl,swell_: swelling-activated Cl⁻ current
IK: intermediate-conductance Ca^2+^-activated K^+^ channels
IP_3_Rs: inositol 1,4,5-trisphosphate
K_Ca_: Ca^2+^-activated K^+^ channels
MAPK: mitogen-activated protein kinase
MEK: mitogen-activated protein kinase kinase
RAF: rapidly accelerated fibrosarcoma
RAS: rat sarcoma
RVD: regulatory volume decrease
RyRs: ryanodine receptors
SOS: son of sevenless
VRAC: volume-regulated anion channel
